# A Rapid and Cheap Method for Extracting and Quantifying Lycopene Content in Tomato Sauces: Effects of Lycopene Micellar Delivery on Human Osteoblast-Like Cells

**DOI:** 10.3390/nu14030717

**Published:** 2022-02-08

**Authors:** Rosario Mare, Samantha Maurotti, Yvelise Ferro, Angelo Galluccio, Franco Arturi, Stefano Romeo, Antonio Procopio, Vincenzo Musolino, Vincenzo Mollace, Tiziana Montalcini, Arturo Pujia

**Affiliations:** 1 Department of Clinical and Experimental Medicine, University “Magna Græcia” of Catanzaro, 88100 Catanzaro, Italy; mare@unicz.it (R.M.); angelo.galluccio@yahoo.it (A.G.); 2 Department of Health Sciences, University “Magna Græcia” of Catanzaro, 88100 Catanzaro, Italy; smaurotti@unicz.it (S.M.); procopio@unicz.it (A.P.); 3 Department of Medical and Surgical Sciences, University “Magna Græcia” of Catanzaro, 88100 Catanzaro, Italy; yferro@unicz.it (Y.F.); arturi@unicz.it (F.A.); romeo@unicz.it (S.R.); pujia@unicz.it (A.P.); 4 Department of Molecular and Clinical Medicine, Sahlgrenska Center for Cardiovascolar and Metabolic Research, University of Gothenburg, 40530 Gothenburg, Sweden; 5 IRC-FSH—Pharmaceutical Biology—Department of Health Sciences, University “Magna Græcia” of Catanzaro, 88100 Catanzaro, Italy; v.musolino@unicz.it; 6 IRC-FSH Department of Health Sciences, University “Magna Græcia” of Catanzaro, 88100 Catanzaro, Italy; mollace@unicz.it; 7 Research Center for the Prevention and Treatment of Metabolic Diseases, University “Magna Græcia”, 88100 Catanzaro, Italy

**Keywords:** tomato sauce, osteoblast, osteoporosis, lycopene, carotenoid

## Abstract

Identifying and quantifying the beneficial molecules contained in nutraceuticals is essential to predict the effects derived from their consumption. This study explores a cheap and rapid method for quantifying lycopene content from a semi-solid matrix. In addition, it compares the in vitro effects of the extracts obtained from different tomato sauces available on the local market with Osteocol^®^, a patented tomato sauce from southern Italy. We performed a liquid extraction of lycopene using suitable solvents. The lycopene extracted was encapsulated in surfactant micelles and finally tested in vitro on Saos-2 cells. The effects exerted by lycopene on ALP and Wnt/β-catenin pathways were investigated by Western blotting. Hexane was found to be the best solvent for lycopene extraction. Spectrophotometrical and HPLC analyses showed similar trends. Osteocol^®^ contained 39 ± 4 mg lycopene per 100 g of sauce, while the best commercial product contained 19 ± 1 mg/100 g. The Osteocol^®^ lycopene extract increased ALP and β-catenin protein expressions in a dose-dependent manner, also showing statistically significant results (*p* < 0.05 respectively). In conclusion, despite both techniques showing similar final results, UV/VIS spectrophotometer is preferable to HPLC due to its cheap, rapid, and accurate results, as well as for the opportunity to analyze lycopene-loaded micelles. The extraction and release of lycopene to bone cells positively influences the differentiation of osteoblasts and increases the expression of the ALP and β-catenin proteins. As a consequence, as a lycopene-rich sauce, Osteocol^®^ represents a useful supplement in the prevention of osteoporosis compared to its commercial competitors.

## 1. Introduction

The Mediterranean Diet (MeDiet) has been promoted worldwide as the healthiest dietary plan. It is characterized by the consumption of high amounts of unrefined cereals, fruit, vegetables, legumes, and olive oil. Tomatoes are the most consumed type of vegetables and represent the highest source of lycopene in the habitual diets of Western countries, including the MeDiet [1].

Lycopene has only drawn the attention of the scientific community only in the last few decades, due both its biological and physicochemical characteristics, as well as for the absence of toxicity and genotoxicity [2,3]. The main health benefits of lycopene are mediated by binding with free radicals, trapping peroxyl-radicals, inhibiting the oxidation phenomena and DNA damage, and stimulating the communication of gap junctions [4,5].

Lycopene is a lipophilic bright red carotenoid pigment responsible for the red color of tomatoes. Due to its powerful antioxidant activity, lycopene reduces the risk and onset of different types of cancers in humans. This carotenoid also exhibits protective or preventative effects in cardiovascular diseases [6], osteoporosis [7], diabetes [8], and several other metabolic disorders related to oxidative stress [9].

Lycopene absorption from foods and supplements is variable and influenced by several inner and outer factors, as well as its concentration in human tissues [10]. In fact, although all-trans lycopene is the most biologically active isoform of this carotenoid, its stability is widely affected by processing and conservation methods, and it can be quickly degraded after exposure to light and oxygen [10]. In addition, the mechanical processing methods of tomatoes, the vegetative stage, and ripening of the berries can influence the percentage of lycopene contained in tomatoes, as well as its chemical stability over time [10].

The functional development of foods and nutraceuticals should thus be based primarily on fresh and high-quality tomato derivatives, such as sauces, ketchup, or tomato concentrate. The high lipophilicity of lycopene limits its administration in aqueous formulations, which underlines the need for novel methods that ensure high bioavailability and blood levels of lycopene, as well as a sufficiently long half-life in the bloodstream. Various efficient formulations and delivery methods are now available that use nano-sized drug delivery systems, including vesicular carriers (liposomes) and micellar suspensions (micelles) [11].

This study describes a novel and reliable methodology for the effective extraction of lycopene from tomato sauces. All the parameters that affect the extraction procedure have been evaluated. The extracts obtained were analyzed using UV/VIS spectrophotometry and high-performance liquid chromatography (HPLC) analysis, and the results obtained with both techniques were compared.

Previous studies have not used highly diluted solutions (conc. < 1 µM) in spectrophotometrical analyses [12], nor compared the spectrophotometer and HPLC instruments. In fact, they have tended to use more expensive and time-consuming approaches than the spectrophotometer, such as Fourier transform infrared (FTIR) spectroscopy [13]. Moreover, they have exploited fixed solvent mixtures, such as hexane/acetone/ethanol, with different molar ratios, without comparing pure solvents in the chemical extraction procedures of lycopene and other carotenoids. Finally, to the best of our knowledge, no studies have investigated the influence of external parameters of the extraction, such as incubation time or the tomato/solvent ratio [14,15].

The method developed was used to compare different tomato sauces available on the market with Osteocol^®^. This is a patented tomato sauce from the south of Italy, obtained from a local and organic cultivar, which is naturally enriched with lycopene [16].

“In fact, studies previously published only paid attention to the comparison between different tomato derivatives, but none investigated just one food matrix in order to provide information capable of bringing health benefits” [15].

The goal of this investigation was to find out more about the possible correlations between food processing and the preservation of beneficial molecules, in addition to determining the feasibility of using foods that are consumed daily, such as tomato-based products, as supplements for medical purposes. A commercial tomato sauce with the highest carotenoid content was finally used for in vitro comparative studies on Saos-2 bone cells, aimed at proving the effectiveness of lycopene in mitigating bone loss. To achieve this target, we designed an innovative and nano-sized drug delivery system (DDS) that makes lycopene more hydrophilic in aqueous media and increases the bioavailability of this carotenoid in the bloodstream.

## 2. Materials and Methods

### 2.1. Material

Lycopene standard, Folin–Ciocalteu reagent, sodium carbonate, gallic acid, sodium nitrite, aluminum chloride, sodium hydroxide, rutin, and L-ascorbic acid were purchased from Sigma Aldrich (Merk, Milan, Italy), as well as 2,2-diphenyl-1-picrylhydrazyl radical powder (DPPH) and all of the solvents used for the extracting and analytic procedures, such as acetone (Ac), hexane (He), dichloromethane (DCM), 2,methyl-tetrahydrofuran (Me-THF), acetonitrile (CAN), and methanol (Me-OH). All other materials and solvents used were of analytical grade. Two sauce bottles for each brand were used (750 mL/bottle) and they were chosen because they were those most used in local market. Bottles of the same brand belonged to the same production lot.

### 2.2. Extraction of Lycopene

The extractions of lycopene from the semi-solid matrix were performed in Pyrex beakers always covered with aluminum foils, in order to prevent light-based degradation. In detail, sauce samples were mixed with an increasing amount of solvents (sauce−solvents ratios of 1:2, 1:5, 1:10, and 1:20) for different time points (10, 60, 120, and 240 min) under continuous stirring.

Supernatants containing lycopene were separated from the semi-solid matrix by separator flasks equipped with a paper filter for organic solvents and were stored in amber glass bottles.

The solvents used for extraction were removed by a rotating evaporator and the obtained powders were re-solubilized in hexane standardized volume for the spectrophotometer and HPLC analyses.

### 2.3. Water Content Determination

The amount of water contained in the sauce samples was determined as previously reported by the Italian Superior Institute of Health in a report regarding the methods of analysis used for the chemical control of food [17].

Briefly, sauce samples were located in aluminum supports and were accurately weighted by analytical balance. All of the samples were incubated in a thermostat-ready oven set at 60 °C in order to promote water evaporation and restrict the carotenoid degradation phenomena. All of the samples were weighed again at different time points and measurements were repeated until a constant weight was obtained in all of the samples investigated, thus suggesting that the water contained in the sauce samples was completely evaporated. The water content (%*w*/*w*) of the sauce was subsequently determined with the following formula:

Water Content (%*w*/*w*) = 100 − [(100 × WF)/WI]

WF is the final weight of the sauce residues; WI is the initial weight of the sauce samples.

### 2.4. Chemical Analyses

Lycopene extracts were analyzed by a UV−VIS spectrophotometer and HPLC apparatus. The spectrophotometer was a Thermo Scientific Genesys 150^®^ equipped with quartz cuvettes. Scan mode was used (wavelength between 190–1100 nm) with a maximum concentration solution of lycopene in order to obtain the absorbance points of the standard solution.

Suitable calibration curves were created with fixed concentrations of standard lycopene (min conc. 0.73 µM and max conc. 11.6 µM). Pure hexane was used as the blank solvent. In agreement with data previously published, different values (~510 and ~472 nm) were used as reference wavelengths in order to quantify and predict the total amount of lycopene and interferences due to the presence of other carotenoids [18].

HPLC analysis was performed according to method described by Olives and coworkers in 2006 [12], with suitable modifications. The chromatographic apparatus consisted of Jasco (Tokyo, Japan) PU-2080plus isocratic pumping system, a Jasco (Tokyo, Japan) AS-2055plus autosampler, a Jasco (Tokyo, Japan) CO-4065 column heater, and a Thermo Separation Spectra Series UV100 (San Jose, CA, USA) UV/VIS detector. For data processing and analysis, ChromNAV software from Jasco (Tokyo, Japan) was used. The analytical column was a µBondapack C18 (300 mm × 2 mm), with 10 µm pore size, with a µBondapack C18 precolumn (20 mm × 3.9 mm). The mobile phase was a methanol/CAN (90/10 *v*/*v*) and it was filtered through a 0.45 µm membrane and degassed prior to use by a Jasco (Tokyo, Japan) DG-4580 degassing unit. The column temperature was 30 °C and the absorbance was read at 472 nm. Peak identification was carried out by a comparison of the retention times with those obtained with standard solution of all-trans lycopene (Supplementary Figures S1 and S2). A suitable calibration curve (min conc. 0.001 mg/mL and max conc. 0.01 mg/mL) was used for quantification of the all-trans-lycopene in sauce samples, as shown in Supplementary Figure S3. The samples (10 µL) were injected into HPLC apparatus with the total acquisition time set at 45 min.

All of the extracts were also characterized in terms of the total phenolic content (TPC), total flavonoids, and antioxidant activity.

Concerning the TPC, lycopene extracts were dissolved in methanol, as previously described in the literature [19,20]. Folin–Ciocalteu reagent (0.25 mL) and 0.05 mL of the extract were mixed and incubated for 5 min at room temperature. Subsequently, 0.5 mL of 20% *w*/*v* sodium carbonate was added to the mixture and incubated for 25 min protected from light. The blue color formed was finally read at 760 nm. A suitable calibration curve was realized using gallic acid (0.1–1.0 mg/mL) as the standard, and the TPC of the extracts was reported as milligram gallic acid equivalent per gram of extract (mg GAE/g).

The flavonoid content was evaluated using the colorimetric assay previously described by Ilahy and coworkers [21], with slight modifications. Briefly, 0.1 mL of the lycopene extracts was diluted with distilled water up of to 1 mL. A sodium nitrite solution (50 µL–5% *w*/*v*) was added and incubated for 5 min. Then, 10% aluminum chloride solution (100 µL) was added. Finally, 1 M sodium hydroxide (0.5 mL) and 1 mL distilled water were added into the mixture and the net absorbance was immediately revealed at 510 nm. The flavonoid content was expressed as milligram of rutin equivalents per 100 g of sample.

The extract antioxidant activity was determined as reported by Aruwa and coworkers [22]. Briefly, 50 μL of each extract and 5 mL of 0.004% (*w*/*v*) solution of DPPH were mixed, vortexed, and incubated in the dark for 30–60 and 120 min at room temperature. Thereafter, the absorbance of each mixture was read with a UV/VIS spectrophotometer at 517 nm. The blank was 80% (*v*/*v*) methanol, and a DPPH solution was used as the negative control while an L-ascorbic acid solution (5 mg/mL) was used as the positive control. The percentage DPPH inhibition was calculated using the following formula:

I(%) = [(A_0_ − A_1_)/A_0_] × 100

A_0_ is the absorbance of the negative control and A_1_ is the absorbance of the extracts/standards.

The percentage radical scavenging activity was normalized as a function of the lycopene and nanoparticle concentrations.

### 2.5. Lycopene Micellar Suspensions

The lycopene micellar suspension was realized by the nanoprecipitation method as previously described in literature [23,24]. Briefly, lycopene extracts were completely dried by a rotary evaporator (Rotavapor^®^, Buchi, Shanghai, China). The obtained powders were solubilized in a low evaporation temperature solvent (acetone) with a proper concentration (2 mg/mL). The aqueous solution was milliQ^®^ water containing 1% *w*/*v* hydrophilic surfactant (Tween 80^®^). The organic phase was added dropwise to the aqueous solution and the obtained mixture was incubated overnight under continuous stirring, in order to promote organic solvent evaporation. The next day, lycopene aqueous suspension was filtrated through 0.45 µm filters with the aim of separating unreacted carotenoids form the micellar suspension. The amount of lycopene concretely entrapped in micelles was estimated by spectrophotometer analyses. All of the procedures were conducted in the dark with the aim to avoid lycopene light degradation.

Micellar suspensions were characterized in terms of the mean size, size distribution, and z-potential using a Zetasizer NanoZS (Malvern Instruments Ltd., Worchestershire, UK), which is a dynamic light-scattering spectrometer with an applied third-order cumulant fitting correlation function. The apparatus was equipped with a 4.5 mW laser diode operating at 670 nm, which was used as a light source for the size analysis, and the back-scattered photons were detected at 173°. The medium refractive index (1.330), medium viscosity (1.0 mPa × s), and dielectric constant (80.4) were set before all experiments. Quartz cuvettes were used for all of the analyses and the samples were diluted 1:50 with a proper medium before the analyses.

### 2.6. Cell Cultures and Proliferation

The Saos-2 cell line was used in the present study as a model of the human osteoblast. Cells were purchased from American Type Culture Collection ATCC (Italy Office, via Venezia 23, 20099 Sesto San Giovanni, Milan, Italy). The cells were maintained in culture with McCoy’s 5A medium added with 15% fetal bovine serum (FBS) and 1% penicillin-streptomycin. The medium was changed about twice a week. With the aim to obtain differentiation in the cell line, Saos-2 cells were incubated with a medium in which FBS was replaced with 10 nM dexamethasone (DEX) in all of the experiments.

In the cell-proliferation assay, Saos-2 cells were cultured in a six-well plate (density 200,000/well). The cells were starved with a serum-free medium containing DEX 4 h before treatment and were finally incubated with 1, 5, and 10 µM micellar suspension containing lycopene extract for 24 h. The exact cellular number was determined by cell count through an optical microscope.

### 2.7. Western Blotting

The Saos-2 cells were cultured in six-well plates and treated as previously explained above. Western blot analysis was performed according to the standard protocol and the mammalian protein extraction reagent (M-PER) purchased by Thermo Fisher Scientific was used for inducing cellular lysis. The following antibodies were used: β-catenin, ALP, and β-actin (company Cell Signaling Technology, Beverly, MA, USA).

### 2.8. Statistical Analysis

The data describe the average of three independent experiments and are reported as mean ± standard deviation. Data concerning all physicochemical properties of the extracts were processed by Microsoft^®^ Excel^TM^ (Office Pack 2016—Microsoft^®^ Corporation, Redmond, WA, USA). Graphs and statistical tests about the correlation between HPLC concentrations and UV/VIS spectrophotometric absorbance were realized with SigmaPlot 12.0 (Systat Software INC, 2011, Chicago, IL, USA). All data related to the in vitro study were analyzed with GraphPad Prism 5.0 software using a two-tailed Student’s *t*-test.

## 3. Results

### 3.1. Lycopene Extraction and Quantification

With the aim to determine the best solvent for lycopene extraction, a reference sauce was incubated of 1 h with different lipophilic solvents and mixtures. hexane (He), dichloromethane (DCM), 2-methyl-tetrahydrofuran (Me-THF), and a mixture of hexane/acetone in 9:1 ratio were used to achieve this target. All samples were analyzed using a spectrophotometer in scan mode, as well as by HPLC. The spectrophotometer analysis allowed for determining the reference wavelength values for lycopene, while HPLC allowed for identifying the exact amount of all-trans lycopene.

All spectrophotometer scansions showed almost identical profiles and two major peaks at wavelengths 472 nm and 510 nm. This last peak was at a wavelength at which the absorbance of β-carotene (and other minor carotenoids) was relatively low and thus caused very little interference [18]. A comparison of the absorbance values suggested that hexane was the best solvent to achieve the most efficacious extraction of lycopene from the sauces (Table 1). In decreasing order, the solvents with a higher lycopene content were He > He/Ac mixture > Me-THF > DCM. In fact, the percentages of lycopene were 17.35, 6.15, 5.25, and 1.20% *w*/*w* (Table 1), respectively.

**Figure 1:**
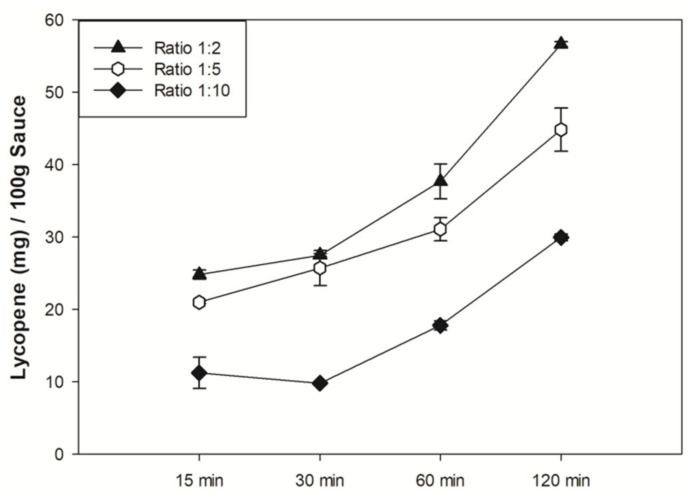
Influence of solvent ratio and incubation time in lycopene extraction. All data are the mean of three different experiments ± standard deviation.

The HPLC analysis confirmed these data and their reliability. In fact, there was a significant correlation between the methods developed by the UV/VIS spectrophotometer and HPLC apparatus (R^2^ = 0.9914; see Supplementary Figure S4). In detail, the all-trans lycopene detected in the extracts was ~0.352 mg/mL for He, ~0.138 mg/mL for He/Ac mixture, ~0.083 mg/mL for Me-THF, and ~0.016 mg/mL for DCM (Table 1). The extracting procedure was performed again with different amounts of hexane and increasing the incubation time, in order to evaluate the influence of both parameters on the lycopene extracting procedures. A sauce/solvent ratio of 1:2 always showed the best results, regardless of the time point considered, and the extraction efficiency (E.E.) always increased in a time dependent manner (Figure 1). As a consequence, 120 min incubation and a 1:2 ratio between sauce (g) and solvent (mL) were finally chosen as the reference settings for the subsequent lycopene extracting process.

### 3.2. Sauces Comparison and Characterization

The developed procedure was used for the efficacious extraction of lycopene from Osteocol^®^ and other commercial sauces, the results of which are listed in Table 2 in decreasing order of this carotenoid as a result of HPLC. The commercial sauce with the highest content of all-trans lycopene showed ~0.329 mg/mL of this carotenoid, thus exceeding 16% *w*/*w* compared to the total extract. The sauce with lowest result had ~0.212 mg/mL of all trans lycopene, which was less than 11% *w*/*w* compared to the extract obtained. Similarly, the all trans lycopene in Osteocol^®^ was ~0.325 mg/mL, which represented almost 16% *w*/*w* of the total extract. All samples were analyzed by UV/VIS spectrophotometer and showed a trend overlapping with the HPLC results (Supplementary Table S1).

**Figure 2:**
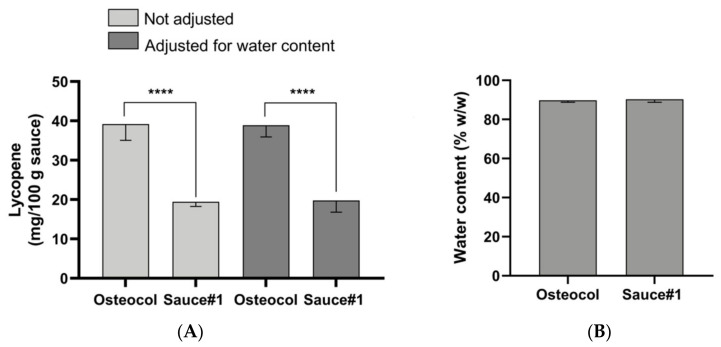
Lycopene content (mg/100 g sauce) in Osteocol and Sauce#1 ((**A**)—**left**) and data adjusted ((**A**)—**right**) as a function of the water content in each sauce (**B**). All data are the means of three different experiments ± standard deviation (Student’s *t*-test **** *p* < 0.001).

The comparison of sauces’ water content produced almost overlapping results. In detail, 100 g of Osteocol^®^ contained 89.75 ± 0.96% water, while the same parameter in Sauce #1 was 90.25 ± 1.49% (Figure 2B). As a consequence, the solid phase in each sauce was also ~10% *w*/*w*.

The comparison of the lycopene mean content between sauces demonstrated that Osteocol^®^ contained almost twice the amount of carotenoid compared to Sauce#1. In detail, for every 100 g of sauce, the mean values were 39.2 ± 4.1 mg and 19.4 ± 1.2 mg for Osteocol^®^ and Sauce#1, thus showing a statistically significant difference (Figure 2A, left, Student’s *t*-test, *p* < 0.001). The statistical significance remained unchanged even when these data were adjusted as a function of the average water content in the sauces, with mean lycopene values reaching 38.91 ± 2.9 mg in Osteocol^®^ and 19.78 ± 2.9 mg in Sauce#1 (Figure 2A, right, Student’s *t*-test, *p* < 0.001). The antioxidant activity of both sauces was evaluated by performing a DPPH assay on the lycopene extracts. Carotenoid samples obtained from both sauces showed a good antioxidant activity, with slight variations due to the differences in term of the lycopene concentration, thus leading to non-statistically significant results (Student’s *t*-test, *p* = 0.21). In detail, the Osteocol^®^ extract (4.25 × 10^−3^ mg/mL lycopene concentration) showed a ~21 ± 0.8% inhibitory ability versus free radicals compared to the positive control, while Sauce#1 (4.85 × 10^−3^ mg/mL lycopene concentration) had ~28 ± 4% as a percentage of the radical scavenging activity. These results were not affected by the encapsulation of lycopene into Tween80^®^ micelles. Indeed, Osteocol^®^-based micelles (concentration 451 µM) had %I value ~20.5%, while Sauce#1-based micelles (concentration 506 µM) reached ~23.4% (Student’s *t*-test, *p* = 0.89). Moreover, the execution of specific tests demonstrated the absence of polyphenols and flavonoids in the sauce extracts (data not shown), as well as preventing the results obtained from being attributable to other macromolecules.

### 3.3. Lycopene Micelles Characterization

Lycopene’s physicochemical properties make it impossible to solubilize the extracts in an aqueous medium. However, performing an in vitro study able to evaluate the effects of our extracts on human osteoblast cells was mandatory, while avoiding the use of toxic solvents such as hexane used for the extractions. For this purpose, we created a biocompatible and biodegradable drug delivery system (DDS) based on Tween 80^®^, which is a non-ionic surfactant widely used in the medical field, with the aim to solubilize lipophilic molecules in aqueous media [25]. The micelles obtained were characterized by their small size (~100 nm), negative *Z*-potential values (~−20 mV), and good polydispersion index values (data not shown).

**Figure 3:**
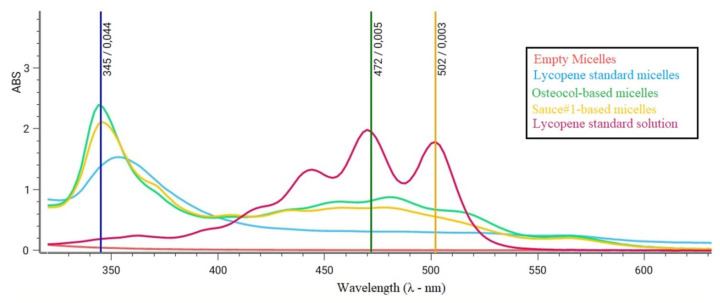
Spectrophotometric scansions between 300 and 650 nm in wavelength. Lycopene standard characteristic peaks were ~472 nm and ~502 nm. Lycopene-based nanoparticles all had a new characteristic peak at ~350 nm, not obtained with empty micelles.

The encapsulation of lycopene in Tween micelles resulted in a shift of the characteristic spectrophotometric signal usually obtained with lycopene (main peak ~472 nm), with the onset of a new and specific peak characterizing the micellar complex between 345 and 350 nm (Figure 3). The intensity of signals obtained at this new wavelength was used for estimating the lycopene exact concentration in the micelles.

### 3.4. Lycopene-Based Micelles Do Not Effect Osteoblast Proliferation In Vitro

With the aim to test the hypothesis that our micellar suspensions containing lycopene might increase osteoblast proliferation, Saos-2 cells were treated with both nano-formulations created up to 24 h.

Cell proliferation was assessed by counting the cells contained in every single treatment well. No statistically significant differences were found in terms of cell proliferation, regardless of the amount of lycopene contained within the micelles as well as within the empty samples (Supplementary Figure S5).

### 3.5. Osteocol-Based Micelles Increased β-Catenin Protein Expression Levels

**Figure 4:**
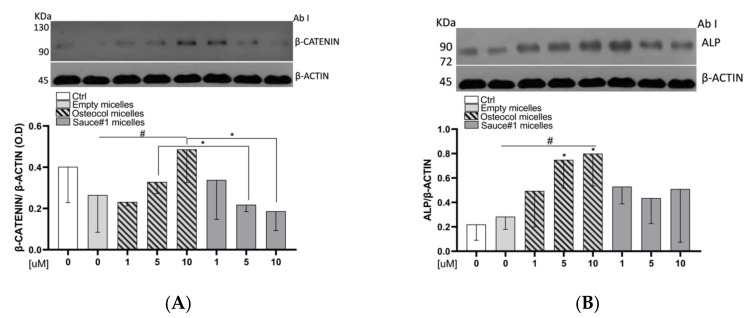
(**A**) Influence exerted by lycopene loaded micelles on β-catenin pathways protein levels; (**B**) influence exerted by lycopene loaded micelles on ALP protein expression. Cell proteins were analyzed by Western blotting with specific antibodies for each pathway. All data are the means of three different experiments ± standard deviation. Statistical analysis: Student’s *t*-test vs. 0 * *p* < 0.05; linear regression ^#^ *p* < 0.05.

Regarding the protein expression, we investigated the influence of lycopene micelles on β-Catenin and the obtained results are shown in Figure 4A. In detail, micelles containing lycopene extracted from Osteocol^®^ positively influenced the β-catenin protein expression in a dose-dependent manner (Linear regression, *p* = 0.04), with the 10 µM treatment exceeding twice the expression seen with the empty micelles. In addition to this, the results obtained with Osteocol^®^-based micelles at 5 µM and 10 µM were also statistically significant compared to the same concentration of Sauce#1 treatments (Student’s *t*-test, *p* = 0.04 and *p* = 0.04, respectively).

### 3.6. Osteocol-Based Micelles Increased the ALP Protein Expression

Nano-sized micelles created with lycopene extracted from Osteocol^®^ almost doubled the ALP expression levels at 5 and 10 µM compared to the empty micelles group (Student’s *t*-test, *p* = 0.03 and *p* = 0.03, respectively). The obtained results showed an increasing dose-dependent trend and statistically significant differences compared to the empty micelles group (linear regression, *p* = 0.017). On the contrary, although the results obtained with Sauce#1 showed an increase in ALP expression, those results were not statistically significant (Figure 4B).

## 4. Discussion

Carotenoid intake is known to be associated with a lower risk of osteoporosis [7,26], and the potential protective effects of lycopene against bone loss have recently been documented [27]. Tomatoes and tomato-based products are rich sources of lycopene and represent over 80% of human food sources, containing different amounts of lycopene depending on the tomato variety [28]. Recently, researchers have been increasingly interested in the effects of lycopene on bone health [29]. In a previous pilot study, we clearly demonstrated that the consumption of lycopene-rich tomato sauce can prevent bone loss in post-menopausal woman, which is also supported by in vitro investigations on human osteoblast-like cells [7].

The extraction of lycopene from semi-solid food matrices can be performed with different solvents and mixtures [10]. However, the best results were obtained with highly lipophilic solvents such as hexane or THF, although the latter solvent provided poorer results due to the higher incubation times, which subsequently increased solvent evaporation under continuous stirring.

HPLC analysis is known to have a higher efficiency and accuracy compared to spectrophotometrical analysis [30]. However, due to advances in new scientific instruments, the results of comparing the HPLC and spectrophotometer in this case made both techniques useful and reliable for quantifying lycopene. In fact, not only was the concentration of lycopene detected by the UV/VIS spectrophotometer and HPLC apparatus similar, but the data obtained by both instruments were strongly correlated (Supplementary Figure S4), and a similar lycopene percentage was found in the extracts (Table 1). This evidence was mainly related to innovations in recent spectrophotometers that are equipped with new and advanced light sources compared to older instruments (xenon vs. tungsten lamps), as well as revolutionary light filters and monochromators, which make them more accurate and sensitive (Abs_MAX_ > 3; wavelength accuracy <0.1 nm).

Carotenoids are known to be sensitive to various degradation processes [31]. In fact, light and oxygen can significantly reduce their average content in food, as well as cooking and incorrect processing or storage procedures [32,33]. In line with findings in the literature, the mean levels of lycopene in Osteocol^®^ were almost double those contained in the best commercial sauce that we used for comparison, with significantly different results also in statistical terms (Figure 2A). In fact, comparing the data described in the literature, tomato sauces and tomato pastes usually never exceeded 15 mg/100 g of product, values largely outdated both from Sauce #1 and from Osteocol^®^ [14,15].

Moreover, despite the fact that the lycopene content was not affected by the sauce water content in our case, all other parameters capable of influencing and often nullifying the beneficial effects of food need to be taken into consideration.

The encapsulation of lycopene in nanosized formulations based on water-soluble surfactants is a successful approach for delivering lipophilic molecules such as lycopene in aqueous media [34], thus preventing the use of organic solvents, which would reduce their biocompatibility and biodegradability.

In fact, similar approaches have already been used both for the delivery of lycopene as well as for other carotenoids, regardless of the delivery systems chosen [35,36]. For example, lipids-based nano-vesicles, such as liposomes, were efficiently used by Zhu et al. to increase the anticancer activity of doxorubicin [37]. Similarly, Yang et al. encapsulated flucoxantin, a marine carotenoid, into polymeric nanoparticles, which were found to increase its anti-Alzheimer effect [38].

In line with the approach used in our study, Xiao et al. recently used different micellar formulations for the efficient delivery of carotenoids [39].

Nevertheless, this approach leads to a change in the physicochemical properties of molecules, which clearly needs to be taken into consideration. In this specific case, lycopene encapsulation in Tween80^®^ micelles led the molecule to interact differently with light sources, with a significant shift in the data obtained by spectrophotometric analyses. This phenomenon can be explained by the formation of a complex due to the interaction between lycopene unsaturations and the oleic acid contained in Tween80^®^, thus explaining the rationale of its new and particular way of interacting with the light emitted by the instrument.

We demonstrated that lycopene-loaded micelles did not affect Saos-2 cells proliferation in 24 h treatments, regardless of the concentrations used and the extraction sauce (Figure 4A). Our findings conflict with data previously published by Kim et al. [40], who found a stimulating effect of lycopene. However, although both projects investigated lycopene effects, the contradictory findings could be attributed to differences between the formulations used, which are characterized by different concentrations, as well as pharmacokinetic and pharmacodynamics parameters [41,42]. Kim et al. analyzed a lycopene rich micro-emulsion, while in this study, we created a suspension-based nano-sized micelles containing a surfactant. Another study conducted on the MC3T3-E1 pre-osteoblast line described lycopene as a cell proliferation inhibitor in 1 µM concentration, with an increased alkaline phosphatase activity [43]. The discrepancy in this case could be attributed to differences in the formulations and cell lines used.

In agreement with data previously published by CK Park, our in vitro studies on Saos-2 human osteoblast cells clearly demonstrated that micelles containing lycopene extracted from Osteocol^®^ increased the protein expression of Wnt/β-catenin and alkaline phosphatase (ALP), both in a dose-dependent manner when compared with a commercial product. Interestingly, the Sauce#1 extract that we used in our comparison did not produce the same effects, thus suggesting that the effects exerted by nutraceuticals are related to the quality of the molecules they contain.

Lycopene is a carotenoid containing 13 double-bonds, which, in their all-trans configurations, provide the most biologically active isoform of this molecule. However, this also makes lycopene highly reactive, and its unsaturations easily undergo oxidation or isomerization processes due to external factors related to processing or storage [28,31]. We thus conclude that short-lasting, high-quality processing, along with appropriate methods of preservation, would lead to a higher concentration of bioactive molecules and therefore be more effective for human health [16].

Nanosciences have revolutionized the methods of delivering molecules. In fact, nano-sized delivery systems, especially micelles, are well known for enhancing the solubilization of lipophilic molecules [25], increasing their blood half-life, as well as increasing and optimizing other pharmacokinetic and pharmacodynamics parameters [44], such as the selective delivery of molecules in specific organs and tissues, or protecting them from degenerative phenomena [45].

Lycopene has already been shown to exert anticancer properties through the Wnt/β-catenin signaling pathway [46,47]. This pathway is important in bone formation, thus playing a key role in the regulation of osteoblast differentiation [48]. Our data confirm that lycopene acts via the Wnt/β-catenin pathway, and that micelles loaded with Osteocol^®^ lycopene provide better results compared to high quality commercial sauces (Figure 4B).

Our results further suggest that Osteocol^®^ sauce is a useful tool for preventing osteodegenerative phenomena, especially considering that ALP is involved in bone matrix formation and is one of the most important biomarkers for studying osteoblastic activity [49].

Unfortunately, the ideal quantity of tomatoes or their derivatives that need to be consumed daily to attain the positive effects of lycopene is currently unknown. Moreover, the lycopene content in tomatoes and tomato-based products varies drastically due to industrial processing and suboptimal storage [50].

Maintaining the natural matrix of food and adding small amounts of fats or oils to the food product may result in a better strategy for obtaining a greater biological response, and thus avoiding the supplementation of food with lycopene [51].

## 5. Conclusions

This study described an effective and efficient procedure for extracting lycopene from semisolid food, and considered all the parameters that could possibly influence the extraction processes. It also demonstrated that UV/VIS spectrophotometry is as useful as other more expensive and time-consuming techniques, such as HPLC, with an almost overlapping data profile.

In addition to this, our results demonstrate, once again, that lycopene has a positive influence on the metabolism of osteoblasts by exerting a clear effect on its differentiation. Data obtained on ALP and β-catenin proteins expression also suggest a plausible role of lycopene in the calcification process. In agreement with data previously published by our research group, these results not only confirm that Osteocol^®^ has beneficial effects on bone deficiencies, but also suggest that Osteocol^®^ sauce is a functional supplement for preventing osteoporosis. 

## Figures and Tables

**Table 1:** Comparison between UV/VIS spectrophotometer and HPLC analyses.

**UV/VIS Spectrophotometer Data**					
**Medium**	**Extract Conc. (M)**	**λ472 nm**	**Lycopene Concentration (M)**	**Lycopene** **(g)**	**Lycopene % (*w/w*)**
DCM	9.31 × 10^−4^	1.65	1.11 × 10^−5^	5.97 × 10^−6^	1.20
2-Me-THF	1.86 × 10^−4^	1.45	9.79 × 10^−6^	5.26 × 10^−6^	5.25
He	4.66 × 10^−5^	1.19	8.08 × 10^−6^	4.34 × 10^−6^	17.35
He/Ac	1.86 × 10^−4^	1.70	1.15 × 10^−6^	6.15 × 10^−6^	6.15
**HPLC data**					
**Medium**	**Extract Injected (mg/mL)**	**Ritention Time (min.)**	**All trans-Lycopene (mg/mL)**	**Lycopene** **(g)**	**All trans-Lycopene (% *w/w*)**
DCM	2	38	0.016	3.12 × 10^−5^	0.78
2-Me-THF	2	38.1	0.083	1.65 × 10^−4^	4.16
He	2	37.7	0.325	6.5 × 10^−4^	16.26
He/Ac	2	36	0.138	2.76 × 10^−4^	6.89

DCM: Dichloromethane; 2-Me-THF: 2-methyl-tetrahydrofuran; He: n-Hexane; He/Ac: 1:1 n-hexane and acetone mixture; mg/mL: milligrams per milliliters; M = molarity; λ = wavelength; g = grams; %*w*/*w* = weight to weight percentage.

**Table 2:** HPLC analyses of the all-trans lycopene contained in sauces.

Sample	Extract Injected (mg/mL)	All-Trans Lycopene (mg/mL)	All-Trans Lycopene (% *w*/*w*)
Osteocol^®^	2	0.325	16.26
Sauce #1	2	0.329	16.44
Sauce #2	2	0.318	15.89
Sauce #3	2	0.295	14.75
Sauce #4	2	0.254	12.7
Sauce #5	2	0.246	12.32

mg/mL: milligram per milliliters; % *w*/*w* = weight to weight percentage.

## Data Availability

Not applicable.

## Supplementary Materials

The following supporting information can be downloaded at: https://www.mdpi.com/article/10.3390/nu14030717/s1, Table S1. Comparison between HPLC and spectrophotometer data in sauces analyses. Figure S1. HPLC chromatogram of pure standard all-trans lycopene. Figure S2. Single HPLC chromatograms of sauces analysis. Figure S3. HPLC calibration curve. Figure S4. Correlation plots between UV-Vis Spectrophotometers Absorbance values and HPLC concentration expressed as intensity. Figure S5. Cell proliferation in vitro test.
