# Supplementary material for: A Rapid and Cheap Method for Extracting and Quantifying Lycopene Content in Tomato Sauces: Effects of Lycopene Micellar Delivery on Human Osteoblast-Like Cells
Source: Nutrients. 2022 Feb 8;14(3):717. doi: 10.3390/nu14030717 (PMC8838682; doi:10.3390/nu14030717)
Supplement: Supplementary file 1 [file nutrients-14-00717-s001.zip › nutrients-1535361-supplementary.pdf]

Chromatogram Information	
User Name	Administrator
Date Modified	1/21/2022 1:38:43 PM
Description	
HPLC System Name	HPLC
Injection Date	11/14/2019 3:17:22 PM
Volume	10.0 [µL]
Sample #	7
Project Name	Lycopene Ros Mar
Acquisition Time	45.0 [min]
Acquisition Sequence	Linearity Lycopene
Control Method	Lycopene extract
Peak ID Table	Lycopene
Calibration Method	Lycopene low
Additional Information	
Column Info	

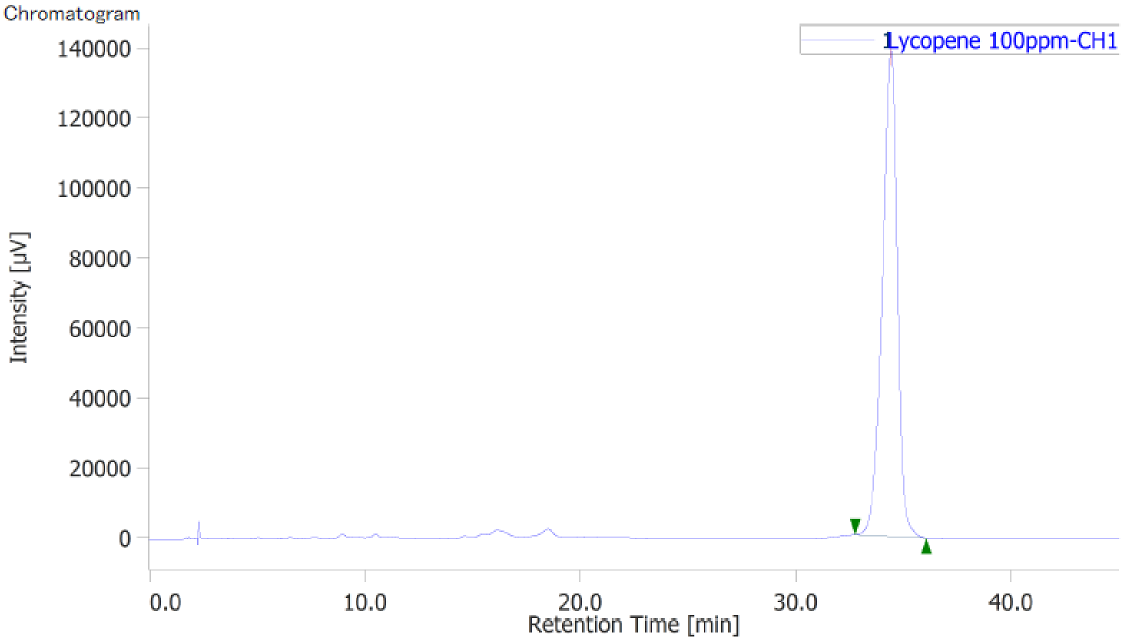

Peak Information

#	Peak Name	CH	tR [min]	Area [µV·sec]	Height [µV]	Area%	Height%	Quantity	NTP	Resolution	Symmetry Factor	Warning
1	Lycopene	1	34.407	6750886	138918	100.000	100.000	N/A	12163	N/A	0.866	
Total				6750886	138918			0.00000				

Figure S1. HPLC chromatogram of pure standard *all-trans* lycopene. Retention time was almost 35 minutes.

A

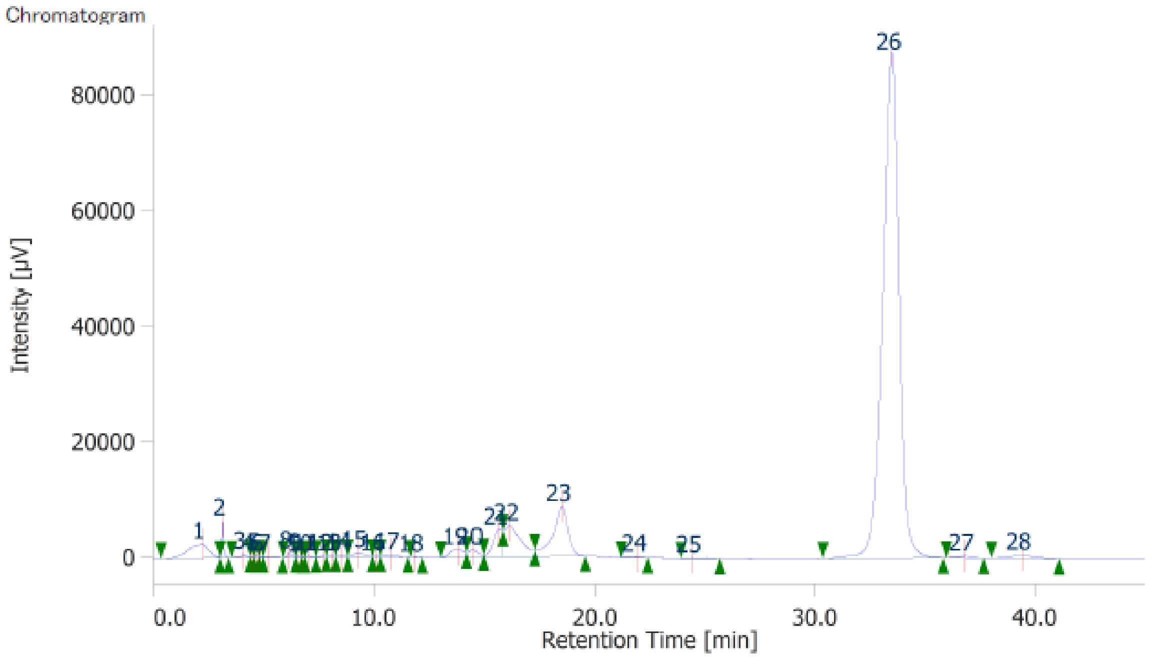

Peak Information

#	Peak Name	CH	tR [min]	Area [µV·sec]	Height [µV]	Area%	Height%	Quantity	NTP	Resolution	Symmetry Factor	Warning
26	Lycopene	1	33.497	4527985	87543	78.177	70.814	70.0873 [ppm]	10458	2.530	0.902	
Total				5791998	123622			70.0873				

B

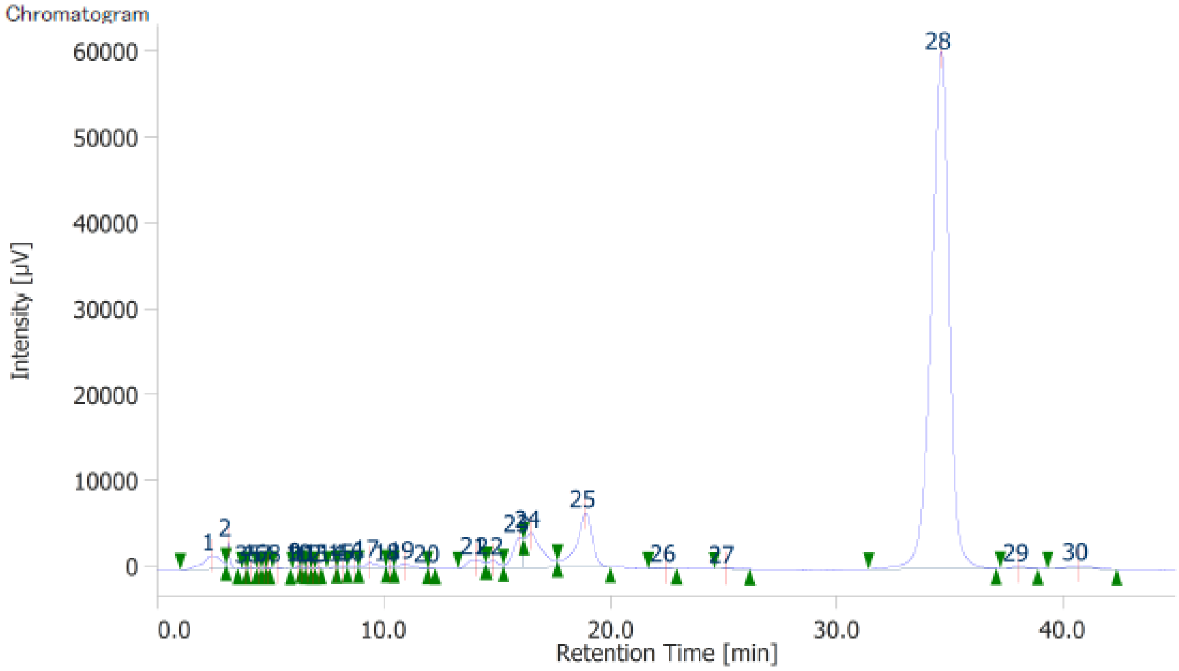

Peak Information

#	Peak Name	CH	tR [min]	Area [µV·sec]	Height [µV]	Area%	Height%	Quantity	NTP	Resolution	Symmetry Factor	Warning
28	Lycopene	1	34.623	3305033	60204	78.578	71.475	51.3322 [ppm]	10057	2.534	0.897	
Total				4206053	84233			51.3322				

C

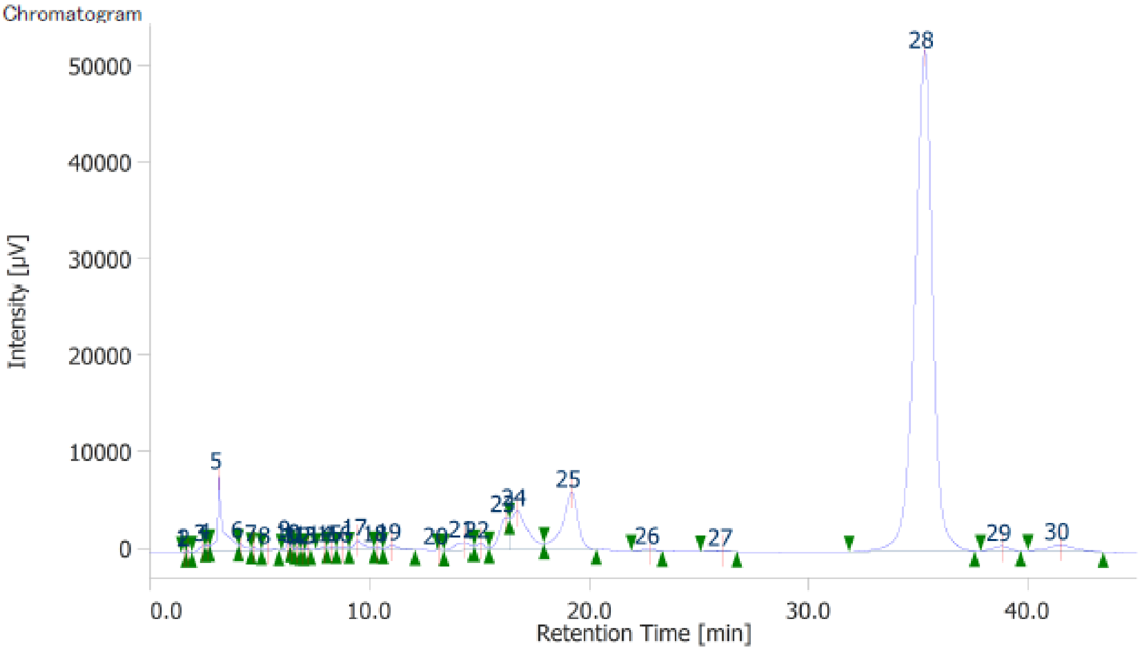

Peak Information

#	Peak Name	CH	tR [min]	Area [µV·sec]	Height [µV]	Area%	Height%	Quantity	NTP	Resolution	Symmetry Factor	Warning
28	Lycopene	1	35.297	2963331	51878	74.720	62.758	46.0919 [ppm]	9943	2.516	0.912	Extrapolation
Total				3965913	82664			46.0919				

D

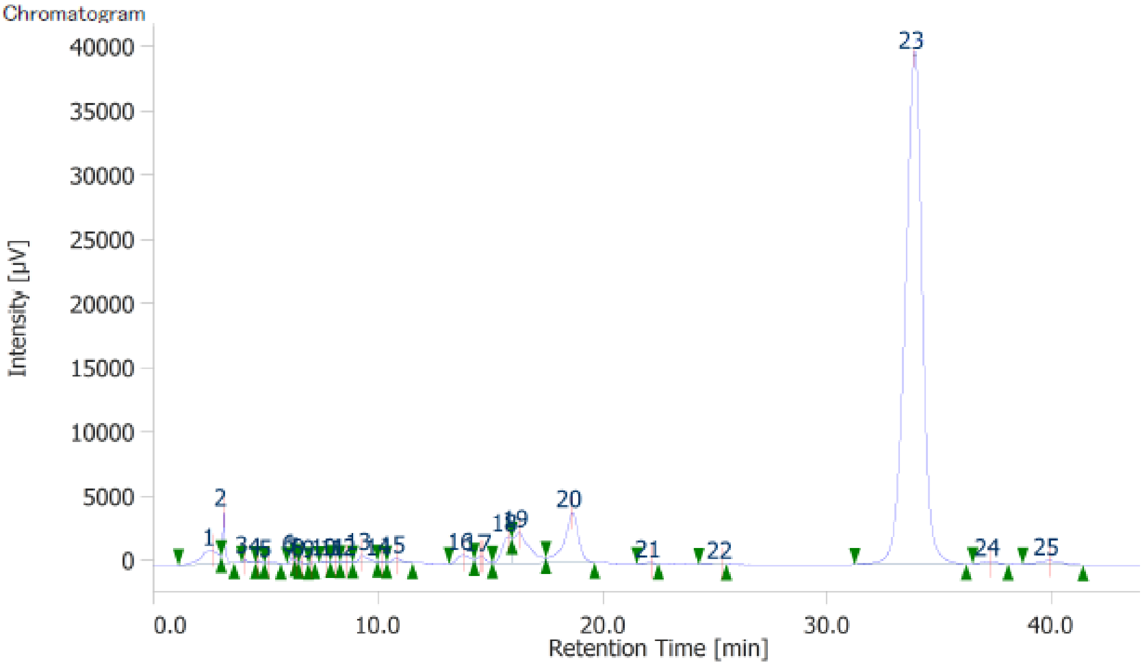

Peak Information

#	Peak Name	CH	tR [min]	Area [µV·sec]	Height [µV]	Area%	Height%	Quantity	NTP	Resolution	Symmetry Factor	Warning
23	Lycopene	1	33.913	2122061	39943	79.294	68.856	33.1902 [ppm]	10145	2.703	0.917	Extrapolation
Total				2676189	58009			33.1902				

E

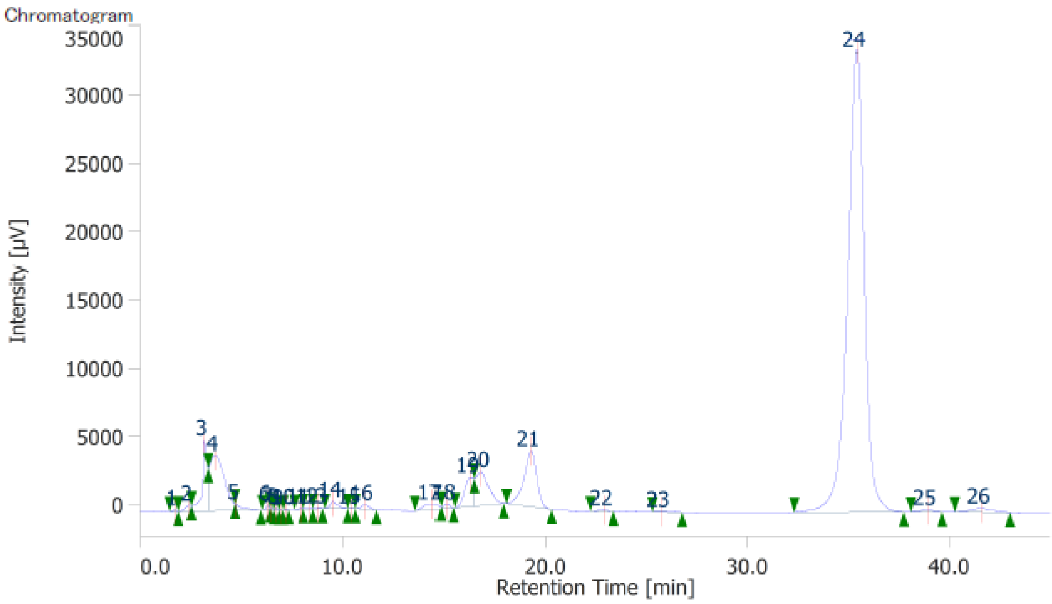

Peak Information

#	Peak Name	CH	tR [min]	Area [µV·sec]	Height [µV]	Area%	Height%	Quantity	NTP	Resolution	Symmetry Factor	Warning
24	Lycopene	1	35.423	1953468	33887	72.085	60.861	30.6047 [ppm]	10005	2.607	0.891	Extrapolation
Total				2709951	55680			30.6047				

F

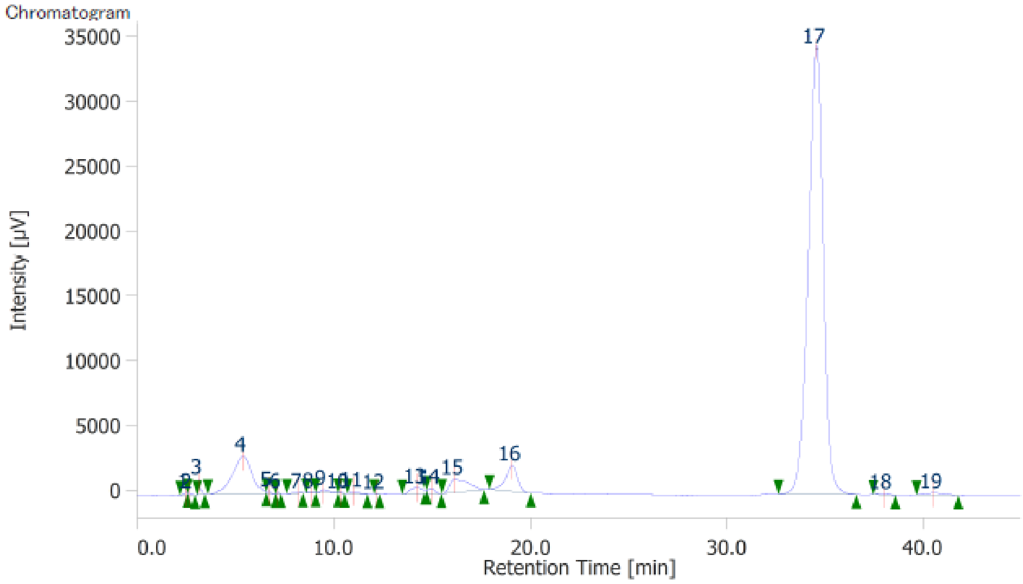

Peak Information

#	Peak Name	CH	tR [min]	Area [µV·sec]	Height [µV]	Area%	Height%	Quantity	NTP	Resolution	Symmetry Factor	Warning
17	Lycopene	1	34.577	1834170	34642	81.569	78.818	28.7752 [ppm]	10233	2.801	0.914	Extrapolation
Total				2248605	43953			28.7752				

Figure S2. Single HPLC chromatograms of sauces analysis. Main peak after ~35 minutes represented *all-trans* lycopene. Data represent in decreasing order of lycopene content Osteocol sauce (panel A), Sauce#1 (panel B), Sauce#2 (panel C), Sauce#3 (panel D), Sauce#4 (panel E), Sauce#5 (panel F).

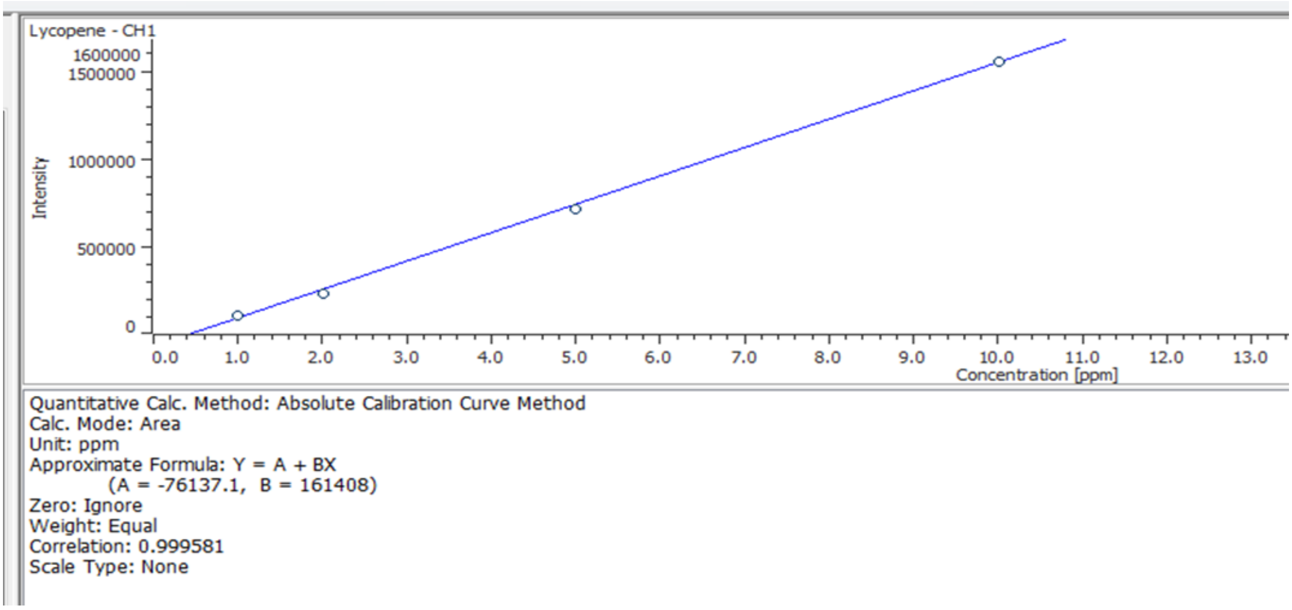

Figure S3. HPLC calibration curve (1-10 ppm concentration).

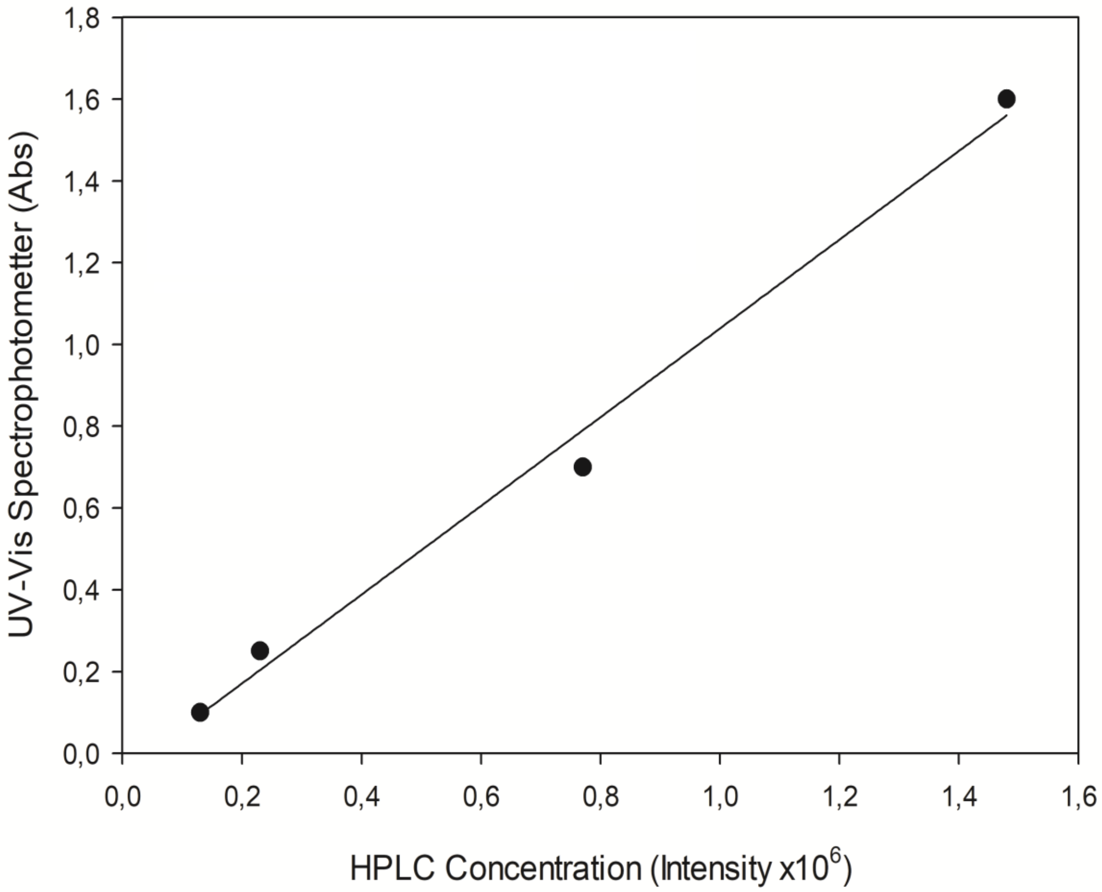

Equation:
f = y0+a*x

R	Rsqr	Adj Rsqr	Standard Error of Estimate
0,9957	0,9914	0,9871	0,0768

	Coefficient	Std. Error	t	P
y0	-0,0458	0,0605	-0,7567	0,5282
a	1,0855	0,0716	15,1563	0,0043

Statistical Tests:

Normality Test (Shapiro-Wilk) Passed (P = 0,1849)

W Statistic= 0,8363 Significance Level = <0,0001

Figure S4. Correlation plots between UV-Vis Spectrophotometers Absorbance values and HPLC concentration expressed as intensity.

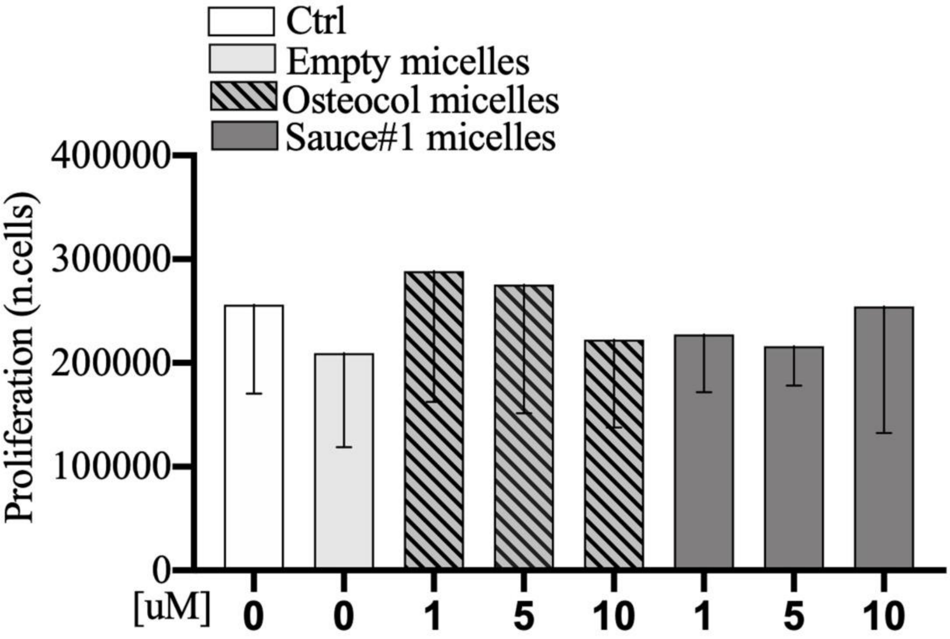

Figure S5. Cell proliferation in vitro test. All data are mean of three different experiment ± standard deviation.

Table S1. Comparison between HPLC and spectrophotometer data in sauces analyses.

Sample 50g/100mL	EXTRACT (mg)	HPLC Data		UV/Vis Spectrophotometer data		
		Lycopene (ppm)	Lycopene %W/W	Abs $_{472\ nm}$	Lycopene (ppm)	Lycopene %W/W
Osteocol®	10.5	325.233	16.26	1.918	385	19.24
Sauce #1	10.1	328.812	16.44	1.725	344	17.19
Sauce #2	21.5	317.845	15.89	1.600	326	16.29
Sauce #3	10.2	295.014	14.75	1.359	276	13.79
Sauce #4	23	254.308	12.7	1.348	274	13.68
Sauce #5	13.9	246.393	12.32	1.159	235	11.75
